# Study protocol of a randomized control trial on the effectiveness of improvisational music therapy for autistic children

**DOI:** 10.1186/s12888-024-06086-3

**Published:** 2024-09-27

**Authors:** A. C. Jaschke, C. Howlin, J. Pool, Y. D. Greenberg, R. Atkinson, A. Kovalova, E. Merriam, I. Pallás-Ferrer, S. Williams, C. Moore, K. Hayden, C. Allison, H. Odell-Miller, S. Baron-Cohen

**Affiliations:** 1 Department of Psychiatry, Autism Research Centre, University of Cambridge, 18B Trumpington Road, Cambridge, CB2 8AH UK; 2 Department of Music Therapy, ArtEZ University of the Arts, Enschede, The Netherlands; 3 Department of Neonatology, University Medical Center Groningen, Beatrix Childrens Hospital, Groningen, The Netherlands; 4 Department of Psychology, Trinity College Dublin, Dublin, Ireland; 5 Anglia Ruskin University, Cambridge Institute for Music Therapy Research, Cambridge, UK; 6 Clinical Trials Unit, Anglia Ruskin University, Chelmsford, UK

**Keywords:** Autism, Music Therapy, Social Communication, Randomised Control Trial, Protocol

## Abstract

**Background:**

Music therapy is the clinical use of musical interventions to improve mental and physical health across multiple domains, including social communication. Autistic children, who have difficulties in social communication and often increased anxiety, tend to show a strong preference for music, because it can be structured and systematic, and therefore more predictable than social interaction. This makes music therapy a promising medium for therapeutic support and intervention. Previous clinical trials of music therapy compared to traditional therapy for autistic children have shown encouraging but nevertheless mixed results.

**Key aims:**

The primary aim is to conduct a randomised controlled trial (RCT) of improvisational music therapy for autistic children and test its effectiveness in at improving social communication and wellbeing, and to reduce anxiety.

**Research plan:**

The RCT will be conducted with 200 autistic children in the UK aged 7 to 11 years old. Participants will be randomly assigned to either improvisational music therapy or support as usual. The trial will be an assessor-blind, pragmatic two-arm cluster RCT comparing the impact of 12-weeks of improvisational music therapy in addition to support as usual, vs. support as usual for autistic children.

**Methods:**

Researchers who are blind to which arm the children are in will conduct assessments and obtain data via caregiver reports. The primary outcome will be the absolute change in the total score of the Brief Observation of Social Communication Change (BOSCC) assessed at baseline, T1 (13 weeks) and T2 (39 weeks) follow-ups. The BOSCC consists of specific items that were developed to identify changes in social-communication behaviours. Secondary outcome measures include: (1) Parent reported anxiety scale for youth with ASD (Note that we do not use the term ‘ASD’ or Autism Spectrum Disorder, because many autistic people feel it is stigmatising. Instead, we use the term ‘autism’) (PRAS-ASD) (2) Young Child Outcome Rating Scale, for wellbeing (YCORS), (3) Strengths and Difficulties Questionnaire (SDQ); and (4) Vineland Adaptive Behaviour Scale (VABS). (5) The Children’s Communication Checklist-2 (CCC-2) will be completed to evaluate pragmatic speech with fluent speakers only; (6) The Music Engagement Scale (MES); and (7) Assessment of the Quality of Relationship (AQR) will be used to evaluate the child-therapist relationships using video-analysis of music therapy sessions. Additional data will be collected by administering the Wechsler Abbreviated Scale of Intelligence (WASI-II), Music at Home Questionnaire (M@H), and children’s versions of the Empathy Quotient (EQ) and Systemizing Quotient (SQ). Audio and video data from the therapy sessions will be collected and analysed (using both human and computer-based feature-coding, e.g., machine learning and AI-driven methods) to identify how music and non-musical interactions foster change throughout the therapy.

**Discussion:**

This study aims to observe if the interactions, engagement, and therapeutic modalities fostered during music therapy sessions can translate to non-musical contexts and improve autistic children’s social communication skills, identifying possible mediating factors contributing to the effectiveness of music therapy, potentially informing policy making and governance.

**Trial registration:**

This randomised control trial is registered with the NIH U.S. National Library of Medicine: https://clinicaltrials.gov/search?term=NCT06016621, clinicalTrials.gov Identifier: NCT0601662, Registration Date 19th August 2023.

**Supplementary Information:**

The online version contains supplementary material available at 10.1186/s12888-024-06086-3.

## Introduction

### Background

Music is deeply-seated in the human mind [1, 2]. Both passive and active listening as well as music-making, can promote bonding, communication, and social understanding [3–5]. There have been several approaches to supporting the development of communication skills, bonding, and social interaction skills and wellbeing in autistic children and music therapy appears to be promising in promoting these [4, 6–9]. Music therapy is the clinical use of music-based interventions to target improvements of mental and physical health across multiple domains [10].

Autistic children, who have difficulties in social communication [11] have shown to have strong preferences for music [12]. This may be because music is systematic and structured and predictable, and autistic children and adults have a strong preference for predictability and patterns [11, 12]. Joint attention and turn-taking are necessary for social interaction and occur naturally in the context of music-making, and are fundamental elements of music therapy [13]. The increased attention and enjoyment that is observed when autistic people are presented with musical stimuli, as compared to verbal stimuli alone, provides a strong rationale for implementing music therapy in autistic children [8, 14]. There is initial evidence that the effects of music therapy are mediated by changes in the brain [15, 16]. Music therapy can have a significant influence on training brain areas such as Broca’s and related speech areas, the frontal and prefrontal cortex associated with executive functions, and deeper structures such as the amygdala, hippocampus and the thalamic-frontal loop associated with wake and sleep cycles [8, 17–19].

While research into the neural substrates of music therapy and autism is still in flux, studies have been applying music therapy to target social skills, using different music therapy interventions including behavioural [8], relationship-centred [20], family-centred [21], social communication-emotional regulation-transactional support (SCERTS) model-based [22, 23], neurological [24], and improvisational [25] outcomes. One of the most well-established and widely used approaches for autistic children is improvisational music therapy [6, 20, 25–28].

### Improvisational music therapy

Awareness of musical parameters and their effect on cognition, emotion and behaviour is essential in music therapy practice. In the UK music therapists are trained at Master’s level and legally registered with the Health and Care Professions Council (HCPC). As the therapeutic medium, music delivered by a trained music therapist supports the therapeutic alliance through providing the framework for verbal and non-verbal interaction, enabling self-expression, and combining intellectual and emotional stimulation [2]. In music therapy interventions, music is used to address a person’s emotional, psychological, social, physical, developmental and functional needs. Music therapists use music and words within techniques and methods that are designed to promote change (ibid.) and improve quality of life. This therapeutic use of music requires a thorough knowledge of music and its potential biopsychosocial effects from a neuroscientific and neuropsychological point of view.

Musical improvisation is a key skill in music therapy practice. Its use in clinical situations allows the music therapist to respond flexibly and meaningfully to the client through the music made in the moment [6, 7, 29–31]. Further knowledge is required about how combinations of music and auditory attributes influence specific psychological and neurological mechanisms in humans, and how pulse, volume, rhythm, melody, harmony, and timbre affect an individual at multiple levels from individual therapeutic change to fundamental neurological development. This gap in knowledge can be bridged through combining clinical improvisational music therapy with behavioural observations, each informing the other in the process of therapy towards addressing the client’s therapeutic goals.

Improvisational music therapy involves the therapist and child spontaneously co-creating music together using singing, playing, and movement. The music therapist follows the child’s interests and focus of attention, which aims to facilitate the child’s social communication development [29]. Crucially, improvisational music therapy is child-led and aims to facilitate a strong sense of personal agency and autonomy. Previous studies have identified that music engagement is more likely to benefit health and wellbeing when the individual has a greater sense of agency in the musical interaction [32, 33]. Similarly, this child-centred developmental approach with autistic children has shown positive effects on social interaction, joint attention, parent–child relationships as well as emotional and motivational development [34–36]. These trials, however, have been limited in methodology and participant classification and selection (see below). There is initial evidence showing that the effects of improvisational music therapy for autistic children are mediated by changes in the brain [37]. This evidence demonstrates that improvements in autistic children’s social communication after improvisational music therapy is related to functional connectivity changes (ibid.). These connectivity changes include greater connectivity in the auditory and subcortical regions, auditory and frontal-motor regions, and lower brain connectivity between auditory and visual regions which are known to be over connected in autism [37]. Improvising with music increases activity in the above mentioned brain regions and the inferior frontal gyrus [38], which is involved in musical (singing) and non-musical (speech) tasks. These regions however, form part of a larger musical neural network, encompassing the whole brain, and recruiting areas overlapping with functions from executive functions through memory and emotion [1, 2]. These networks are in turn activated when improvising, and this is associated with the (de)activation of the dorsolateral prefrontal cortex and the balance between activation and deactivation of neural networks [2, 6, 15, 39, 40]. Oxytocin [41, 42], dopamine [43], noradrenaline and epinephrine[43, 44] are released when listening and playing music, with oxytocin and dopamine increasing when group singing, performing and when improvising. [45, 46]. These same networks are implicated in social processes, such as mentalizing, empathy, and synchrony, which are all components involved in social connectedness [17, 47].

Music therapy may be more appealing and comfortable for autistic children than other treatment methods (e.g., verbal communication-based therapies [25]), because music therapy may be less confusing than spoken language and more predictable than social engagement (i.e., psychotherapy or cognitive behaviour therapy). Embedded in music therapy is the potential for prediction and anticipation brought about by musical structures, which have neural links to motivation and reward [43, 44]. However, prior research on this topic has relied too heavily on small-*N* studies, case studies, and non-RCT clinical trials. Thus, there remains a lack of evidence about what the enduring effects of music therapy are on social interaction, communication, and related skills [36], and a lack of evidence about the dose–effect relationships. Further evidence is also needed to establish whether the positive effects observed within therapy sessions generalise to other situations (i.e., interaction and communication with other people). It is also unlikely there will be a single therapy which is beneficial to all autistic people. Therefore, additional research is needed to establish which forms of support are most suitable for whom.

### Music therapy and autism

Music therapy is an established allied health profession in the UK, the US, Canada, Europe and Australia. Previous autism treatment trials of music therapy compared with traditional therapeutic approaches have shown encouraging but mixed results [4–6, 34, 36]. The recently updated Cochrane review by Geretsegger et al. [36] pointed out that these trials have been limited in several ways. First, some have relied on small samples and included children with wide age ranges. Second, the primary outcome of reduced autistic symptom severity was measured using instruments such as the Autism Diagnostic Observation Schedule (ADOS) [48] that are not sensitive enough to detect comprehensive changes in social communication [49]. This limitation was present in a recent large-scale, multi-centre, pragmatic RCT [6] of improvisational music therapy. In this trial, Bieleninik et al. found no significant difference between the intervention and control groups in symptom severity based on the ADOS social affect domain over a period of five months. It concluded that the findings do not support the use of improvisational music therapy for symptom reduction in autistic children. However, their study reported substantial variations in how the music therapy protocol was implemented at different sites, making it difficult to interpret the results conclusively.

These findings are in contrast to the updated Cochrane meta-analysis by Geretsegger et al. [36], which showed a moderate positive effect of music therapy with autistic people on “total autism severity and quality of life and probably does not increase adverse events immediately after the intervention” ([36] p. 34). However, this meta-analysis utilised broad inclusion criteria, which resulted in a heterogeneous population of possible responders and non-responders, thereby reducing the likelihood of significant group differences on the defined primary outcome [49]. The findings from Bieleninik et al.’s RCT are also in contrast to a large volume of research of case–control, and longitudinal studies showing that music is effective in targeting social behaviours for autistic people [30, 35, 50]. For example Kik et al. [27] reported more instances of ‘joy’ and ‘emotional synchronicity’ when autistic children are engaged in improvisational music therapy as compared to just play. Therefore, there is some evidence in support of the use of music therapy for autistic children, however, what is needed is a well-powered RCT that overcomes limitations and methodological constraints in prior research. Given that music therapy has the potential to improve the lives of autistic individuals and their families, especially in relation to wellbeing and improved adaptive functioning, the field is in urgent need of rigorous scientific research to provide evidence of these benefits. It is important that interventions target outcomes that autistic people want support with, such as mental health, rather than targeting autism itself, since autism per se is part of the person’s identity. Music therapy is designed to supplement rather than replace other interventions for autistic children.

## Method

### Ethics and registration

Here, we describe a new study of improvisational music therapy for autistic children, and report the protocol before the study is complete. Researchers will assess eligibility to participate, prior to collecting baseline assessments and demographic data. Those who are ineligible will be thanked for their time and informed of the reason(s) for this. The process of obtaining informed consent will be conducted in accordance with the requirements of Research Ethics Committee guidance and Good Clinical Practice, where possible child consent will be collected alongside parental consent. This research has been approved by the Cambridge Psychology Research Ethics Committee. This randomised control trial is registered with the NIH U.S. National Library of Medicine: https://classic.clinicaltrials.gov/ct2/show/NCT06016621, clinicalTrials.gov Identifier: NCT06016621, Registration Date 19th August 2023.

### Aims

The primary aim is to conduct a trial of improvisational music therapy for autistic children. There are five overarching objectives:

1. To examine whether improvisational music therapy in addition to support as usual is superior to support as usual alone in improving social communication skills in autistic children (primary measure).
2. To examine whether the therapeutic relationship predicts the development of social, communication and language skills among autistic children (primary measure).
3. To determine whether improvisational music therapy in addition to support as usual is superior to support as usual alone in improving wellbeing, and reducing anxiety, in autistic children (secondary measures).
4. To examine whether improvisational music therapy in addition to support as usual is superior to support as usual alone in improving adaptive functioning in autistic children (secondary measures).
5. To examine whether improvisational music therapy in addition to support as usual is superior to support as usual alone in reducing psychosocial problems in autistic children (secondary measures).

## Population

### Inclusion criteria

The aim is to recruit 200 children.

(i) who are: aged 7 years 0 months to 11 years 11 months, over a 30-month period;
(ii) who have a clinical diagnosis of autism made by a qualified professional according to ICD-10 criteria. The diagnosis will be confirmed by requesting a copy of the clinical report detailing the diagnosis or verified verbally by the child’s parents. Reported diagnosis has been shown to be highly correlated to independently verified diagnosis [51].
(iii) whose parents/guardians provide informed consent for their children to be enrolled in the trial;
(iv) whose parents/guardians are willing for the music therapy sessions and BOSCC assessments to be video recorded for monitoring and research purposes;
(v) whose parents/guardians must be willing for their child to attend two music therapy sessions per week for the duration of the trial;
(vi) who have any level of expressive language, from those who are minimally verbal to those with fluent speech.

### Exclusion criteria

Children will be excluded if:

(i) they have received regular individual music therapy in the last year as this would be likely to have a strong influence on the course of therapy;
(ii) they have a severe hearing deficit as this would alter the aim, course and implementation of therapy;
(iii) if caregivers are unable to attend for the psychological assessments with their child;
(iv) if caregivers do not have a basic understanding of English.

### Power calculation and sample size

A total sample of *N* = 200 children is required. The proposed sample size is based on power analyses and similar RCTs of early intervention in autism using a range of treatment methods and similar outcome measures [4, 52–54]. Specifically, we conducted a power analysis in G*Power, which estimates that a total sample of 200 (*N* = 100 in each group) is needed to have a 95% probability of detecting a small effect (*d* = 0.115) using a repeated measures F-test to compare change over time. We selected a small effect size based on four previous music therapy assessor blinded clinical trials that reported effect sizes that ranged from *d* = 0.11 to 0.23, for the outcome of social interaction [4, 5, 53, 54]. Loss to follow-up in trials of psychosocial intervention for autistic children tends to be low. The PACT trial of parent mediated communication focused treatment reported a follow-up rate of 96% at 13 months [53]. In contrast, the TIME-A randomised clinical trial reported 14% of children were lost to follow up at 5 months [52]. Accordingly, to account for a participant attrition rate of 14% we will continue to recruit participants to the study until 200 participants have completed the baseline and endpoint assessments.

## Feasibility and procedure

### Recruitment

Participant recruitment and intervention is taking place primarily in schools. Recruitment involves the research team contacting primary schools (mainstream and special) in the UK. Information about the research is sent to all schools located in the recruiting areas, inviting them to participate with instructions on how to contact the research team if they would like further information, have any questions, or would like to express interest. This is followed up by a phone call to discuss the study with an appropriate staff member at the school. An email with basic information about the study and contact details for the research team is sent out by the school to all parents. Parents or guardians of children aged 7 years 0 months to 11 years, 11 months who have a clinical diagnosis of autism are invited to contact the research team directly or inform a member of staff in the school that they would like to be contacted by the research team.

### Informed consent

Parents who express an interest in taking part in the research will be invited to a meeting (face to face or online where necessary). At this meeting the parents are provided with written and verbal information about the study including a participant information leaflet. The researcher encourages parents of potential participants to spend as much time as they want asking questions about the study and considering whether they and their child would like to take part. In all instances potential participants have at least three days before deciding whether they wish to take part in the study. Participants and parents can opt out of the study at any time without having to provide a reason for their withdrawal.

If families want to take part but are unable to attend their school for the in-person assessments, they are given the opportunity to complete the in-person assessments at home. All parents are invited to take part in the school setting in the first instance. If research assistants need to visit participants homes, they will attend as a pair to ensure their safety.

### Rate of recruitment

**Fig. 1:**
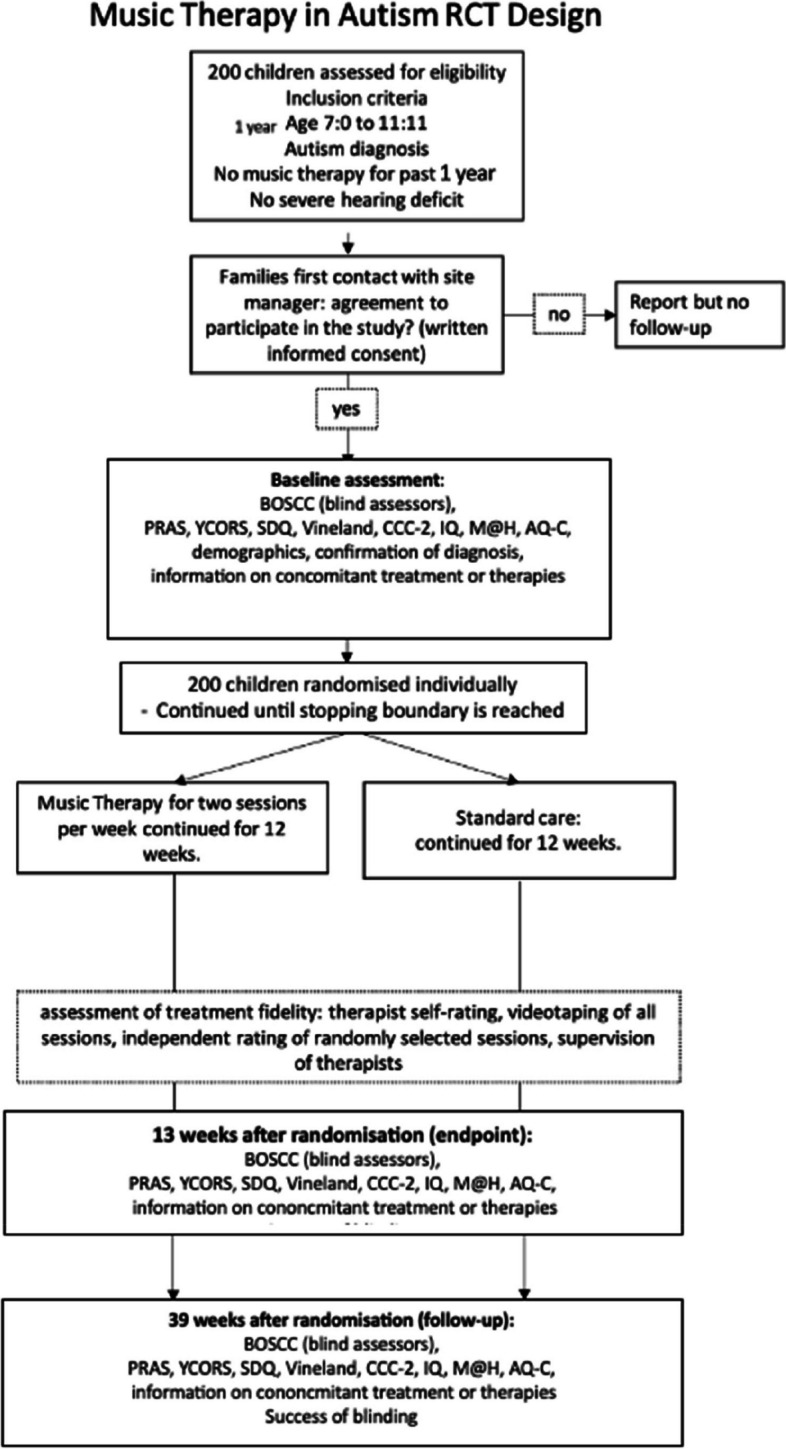
Protocol design flow diagram

Four full-time research assistants will recruit participants from schools during this period. A drop-out rate of 14% is expected based on the results of previous studies. To take account of drop out we will continue to recruit participants until a minimum of 200 have completed the baseline Fig. 1.

### Randomisation

Eligible participants are randomised using the Sealed Envelope Ltd randomisation software for clinical trials after all baseline measures are complete. Enrolled participants will undergo 1:1 block randomisation, to either support as usual plus improvisational music therapy sessions (intervention arm) or support as usual (control arm). Participants will be stratified based on the version of the BOSCC that they are allocated (i.e. (1) Minimally Verbal, (2) Phrase Speakers, or (3) Fluent Speakers, so that there are equal numbers in each group, and that the control group and intervention group are balanced. The randomisation system will store the pre-determined sequence of randomisation. This list is not available to the investigator [or ARCTU staff]. Once a participant has consented to take part in the trial, designated members of the research team will log in to the web based Sealed Envelope Ltd software. After entering information required to check eligibility, participants will be randomly allocated to either arm.

### Design

The trial is an assessor-blind, pragmatic two-arm RCT comparing the impact of adding improvisational music therapy to support as usual for autistic children over a 12-week period. The setting and target organisations are mainstream and special schools in the UK. Blinding of participants is not feasible due to the nature of the intervention. Research assistants collecting outcome data will be blinded to which arm the children are allocated to. Instances of unblinding will be recorded using a case report form (CRF) (which will include information on who was unblinded, the source of unblinding and the reason for unblinding). Parents and teachers will be aware of the treatment allocation for the trial and will be encouraged and reminded not to reveal the allocation to the research assistants.

#### Data collection

Baseline data will be collected prior to randomisation T0 (to establish eligibility to participate and assess baseline social communication skills and wellbeing outcomes), at the primary endpoint T1 (13 weeks after randomisation), and the secondary endpoint T2 (39 weeks post-randomisation and 26 weeks after the end of music therapy). T0 will take up to 2 h and 20 min. T1 and T2 will take up 1 h and 30 min. These time estimates include set-up times and have been included in the participant information leaflet.

## Materials

### Primary outcome

#### Brief observation of social communication change (BOSCC)

The BOSCC consists of specific items that were developed to identify changes in social-communication behaviours over relatively short periods of time by quantifying subtleties in both the frequency and the quality of specific behaviours. It measures absolute change in social-communication, reflected in the total score [55] assessed at baseline and the primary endpoint. It was developed by modifying and expanding codes from the ADOS-2 [56]. There are three versions of the BOSCC: one for individuals who are minimally verbal (MV – read less than 30 words), one for those with phrase speech/young fluent speakers (PS/YF up to age 6—8) and one for fluent speakers (F1/F2 with two sets of materials, one for children and one for adolescents and adults). The BOSCC is flexible, easy to code, and minimally-biased by caregiver or clinician report. It can be used across a variety of settings (e.g., across multi-site studies, in clinics or at home) and coding does not require a highly experienced or credentialed coder. Scores are based on the observation of social communicative behaviour during naturalistic interactions between a child and an adult, rather than a standardized semi-structured professional–child interaction. The scoring scale of the BOSCC allows the capture of nuanced variations in behaviour, with a score range of 0–5, with higher scores being indicative of more atypical behaviour. To avoid bias in observation and assessment, assessors administering the BOSCC will be blinded to the child’s group allocation. Success of blinding will be verified by asking assessors whether they inadvertently found out about the child’s allocation. Additionally, raters of the BOSCC videos will be blinded to the timepoint at which each video was taken. One advantage of the BOSCC is that direct observation reduces the risk of responder or acquiescence bias likely for caregiver or clinician report measures such as the Clinical Global Impressions (CGI) [57].

##### Secondary outcomes and participant characteristics

1. The Music Therapy Communication and Social Interaction scale (MTSCI) [58]. The MTSCI evaluates communicative and socially interactive responses that are elicited during music therapy sessions and is completed by the music therapist.
2. The Assessment of the Quality of Relationship (AQR) to measure the association between the therapeutic relationship and the development of social, communication and language skills in children allocated to the music therapy intervention[59] as completed by the music therapist.
3. Strengths and Difficulties Questionnaire[60], a brief behavioural questionnaire suitable for children aged 3–16 years old, which will be administered at T0 – T2.
4. Vineland Adaptive Behaviour Scale[61], a well-validated measure of adaptive functioning, which will be administered at T0 – T2.
5. Children’s Communication Checklist-2 (CCC-2)[62], a parents self report measure designed to assess the communication skills of children 4 to 16, which will be administered at T0 – T2.
6. Children's subjective wellbeing measured on the Child Outcome Rating Scale (CORS) or the Young Child Outcome Rating Scale (YCORS). The CORS is a well validated[63], four item measure designed to assess areas of life functioning which include personal wellbeing, interpersonal wellbeing, social wellbeing, and overall wellbeing in children aged 6 – 12. Administered at T0 – T2.
7. Music@Home Questionnaire. Parents will also be asked to report on the child's musical environment at home and their own attitudes in relation to the importance of music to wellbeing. This questionnaire is part of the demographic intake for the study (T0). Politimou, N., Stewart, L., Müllensiefen, D., & Franco, F. (2018). Music@ Home: A novel instrument to assess the home musical environment in the early years. *PloS one*, *13*(4), e0193819.
8. Parents-Rated Anxiety Scale (PRAS-ASD) is a self-rapport-parent-rated assessment tool to capture participants’ anxiety levels [64]. Administered at T0.
9. The child’s level of verbal and non-verbal intelligence, which will be assessed using two subscales of the Wechsler Abbreviated Scale of Intelligence (WASI-II) [65–69]. Administered at T0.
10. Parents/guardians will be asked to complete the child version of the Autism Spectrum Quotient (AQ-C) [70] at T3.
11. The children's versions of the Empathy Quotient (EQ-C) and Systemizing Quotient (SQ-C), referred to as Child EQ-SQ [71, 72], at T3 will be used to observe how E-S cognitive styles moderate or mediate the responses to music therapy in both groups.
12. In addition, standard demographic parameters (gender, detailed age, handedness, first language, family size, parents’ educational background), co-occurring conditions i.e. mental and or physical health, and information on concomitant treatment and therapies will be recorded.

### Treatment intensity

The trial involves one active treatment group in which 24 music therapy sessions are delivered twice a week over the 12-week treatment period. As this is a pragmatic trial, it is necessary to account for school holidays and unexpected absence of participants. Therefore, the minimum number of sessions each child will need to attend for treatment adherence is one per week for six weeks, and the maximum duration over which 24 sessions may be offered is sixteen weeks.

### Intervention

Participants will be randomly assigned to either the intervention or the support as usual control group. This trial will examine the effectiveness of improvisational music therapy [73]. Music therapy sessions will last for 30 min (equating to one hour of therapy time). Sessions will be delivered twice weekly to the child individually, at their school. Music therapy sessions will be provided by academically trained Health and Care Professions Council (HCPC) registered music therapists in the UK (master’s level or equivalent) with clinical experience of working with autistic children. Considering the time required for preparation, conducting, and writing up the session, each therapist will allow 2 h per child for each session. Each child in the intervention arm will receive 24 sessions of music therapy over a 12-week period.

During the session, the therapist engages with the child by playing and sharing musical instruments, and/or sings while being attuned to the child’s behaviour and expression. Various improvisational techniques are employed to engage the child. There are opportunities for pulse, rhythmic, dynamic or melodic patterns, and timbre to be mirrored, reinforced, or complemented, which allows for moments of synchronization between the therapist and the child, giving the child’s musical expressions a pragmatic meaning within this context. The therapist may also gently musically challenge the child by interrupting or changing expectations or patterns that have been jointly developed, in order to elicit specific social communication behaviours. Further, there are opportunities for the child to develop and enhance social communication skills such as joint attention, sharing affect, reciprocity, shared history, scaffolding, imitation, and turn-taking. These have been shown to develop social competency [74] and also resilience [75] (see supplementary material 1 for an overview of IMT principles).

### Treatment fidelity

To determine whether treatment is delivered as intended, all music therapy sessions will be videorecorded to allow assessment by trained, independent assessors who will not be involved in collecting outcome data or providing the intervention. Recordings will be made with participants who have given informed consent. To provide an assessment of fidelity of intervention delivery, 20% of the recorded sessions will be reviewed and rated according to a fidelity checklist by the independent observers. Inter-rater reliability will be calculated for a subsample of sessions. Adherence and competence will also be monitored and sustained through clinical supervision.

### Support as usual

Participants allocated to ‘support as usual’ receive usual care only. They will receive support as usual from their general practitioners (GPs), mental health and education/allied health professionals or any other care as usual they have received prior to the trial. Support as usual is defined as normal practice for each school in addition to the usual support from the specialist teaching teams for autism in the area. They will not receive the music therapy intervention or any extra support services from the research team. Any concomitant treatment or therapeutic interventions that participating children might receive will be recorded during assessment sessions before randomisation and following the primary endpoint, specifying the kind and amount or frequency of intervention. Control participants are asked not to undertake any music-based therapy groups, sessions or related therapies for the duration of the trial. Should a participant in the control arm wish to withdraw from the study – particularly in the case where music therapy becomes available to the participant, the same process used for intervention participants will be employed.

### Data analysis

Primary and secondary outcome measure will be compared from baseline across 1st and final follow-up (T0 – T2). Due to the multitude of data, the primary outcome will be analysed with a multi-level analysis, incorporating secondary outcome measures using R-statistical software. This approach will allow to focus on both mediators as well as moderators from intervention to outcome effects. Differences over time will be analysed using a repeated measures F-test, and incorporated into the multi-level analysis, to indicate changes between groups over time between T0, T1 and T2.

The primary analysis will utilise a modified intention-to-treat principle (ITT) [76, 77] that includes all participants whose data were collected across T0-T2. For analysis of the primary outcome measure, (BOSCC), after assessing the data for normality, BOSCC scores will be standardized and analysed using a generalized linear mixed model to evaluate change over time for both the music therapy group and the control group. Age, gender, IQ, autistic traits, cognitive style and musicality (as measured by the AQ-C and Child EQ-SQ, and M@H) will be entered as covariates and tested for interaction effects. The secondary outcome measures will also be analysed using generalized linear mixed models.

Audio and visual data collected from the music therapy sessions will be coded by assessors utilizing a standardized protocol that can later be assessed for inter-rater reliability. Audio data will also be analysed using machine-learning and AI driven computer feature extraction. Specifically, non-musical spoken-language will be sectioned out using an open-vocabulary approach [78]. The musical elements of the session will be coded using the ESSENTIA software library [79] to extract low-level audio features (e.g., loudness, speed, and density), and high-level music features (style features such as mellow, intense, sophisticated, and mood features such as joyful, sadness, depth, calming). ESSENTIA has been used successfully in prior research across audio stimuli sets and its computer-based AI ratings of music features been shown to mirror human perception of them [80–82].

## Discussion

This randomised control trial protocol aims to address several active challenges in the autism intervention field. It addresses the desire of both autistic children and parents who welcome a creative and non-invasive and non-pharmacological intervention. It will also contribute evidence to the current international discussions around the importance of music therapy. This trial will involve autistic children in early school years, thereby, addressing the need for interventions for an under-researched cohort. Most evidence-based interventions focus on preschool aged children and only a handful of autism intervention RCTs have included school age cohorts and demonstrated sustained and evidence-based effects. Finally, this trial will implement a rigorous methodology and an objective primary outcome measure to capture autism-related outcomes. Furthermore, we will capture developmental, cognitive, emotional, and behavioural changes. These metrics will enable wide-ranging testing of the effectiveness of improvised music therapy for autistic children and their families.

### Potential impact of the project

Autistic children and their families will be most impacted by the results of our study. The results will be disseminated with policy makers, care givers, teachers, and parents, and will furthermore be communicated with public and health organisations, who are responsible for the recommendation of treatment and the development of guidelines for autistic people, e.g. the National Institute for Health and Care Excellence (NICE) Guideline Development Group for Autism, as currently there are no clear guidelines for music therapy for autistic children.

The need for rigorous research on music therapy interventions in autism has been highlighted by several leading organizations. For example, the National Institute of Health’s (NIH) Sound Health Initiatives has raised $20 million over five years to research music as a way to treat a wide range of conditions. One of their primary goals is to “study musical rhythm synchronisation as a mechanism of health social development and how that is disrupted in children with autism spectrum disorders, with the goal of developing music interventions for social communication” [83]. Accordingly, our RCT on the effectiveness of improvisational music therapy will provide a significant advance to the much-needed understanding of how music therapy can help autistic children and their families.

### Future studies to achieve client benefit

Findings from our study will provide groundwork upon which future research can be built. We plan to conduct future RCTs on adult autistic populations. We also plan to extend our RCT based on one-to-one individual therapy to groups but conducting RCTs of group music therapy in autistic children and adults. Furthermore, we aim to conduct future studies incorporating hyper scanning that can measure the role of brain-to-brain synchrony between clients and their therapists during the intervention. In addition, we plan to explore the avenues of potential hypersensitivity and hyperacusis in children born extremely or very preterm and with early diagnosed autism. Here, early music interventions in the neonatal intensive care unit, hold potential benefits for minimising hypersensitivity and hyperacusis. Overall, in this research program we aim to establish if music therapy improves wellbeing and social communication skills in autistic children.

## Supplementary Information

Supplementary Material 1.

Supplementary Material 2.

Supplementary Material 3.

## Data Availability

Data sharing is not applicable to this article as no datasets were generated or analysed during the current study.

## Data availability

No datasets were generated or analysed during the current study.
