# Supplementary material for: Study protocol of a randomized control trial on the effectiveness of improvisational music therapy for autistic children
Source: BMC Psychiatry. 2024 Sep 27;24:637. doi: 10.1186/s12888-024-06086-3 (PMC11437930; doi:10.1186/s12888-024-06086-3)
Supplement: Supplementary file 1 — Supplementary Material 1. [file 12888_2024_6086_MOESM1_ESM.pdf]

UNIVERSITY OF CAMBRIDGE
Autism Research Centre

a.r.u. Cambridge Institute for Music Therapy Research

Autism-CHIME
Children's Improvisational Music Therapy Evaluation

Prof Simon Baron-Cohen
Director, Autism Research Centre
Department of Psychiatry
University of Cambridge

Dr Jonathan Pool
Senior Research Associate
Anglia Ruskin University

CONSENT FORM

Title of Project**: Improvisational Music Therapy for Autistic Children [Autism-CHIME]**

Trial Registration: NCT06016621

Principal Investigators: Prof. Simon Baron-Cohen and Dr Jonathan Pool

Please initial boxes

I confirm that I have read and understand the information sheet (dated 20th July 2023, version 2.2) for the above study. I have had the opportunity to consider the information, ask questions and have had these answered satisfactorily.	☐
I understand that my child's and my participation is voluntary and that we are free to withdraw at any time, even after signing consent, without having to give any reason, without my medical or social care or legal rights being affected.	☐
I understand that the data will be pseudo-anonymised until the end of the data collection period, expected in 2026, at which time it will be fully anonymized. It will not be possible for participants to withdraw their individual data after it is anonymised.	☐
I understand that the data (i.e. information about my child and I) that I give to the research team during the study may be looked at by individuals from the research team and Anglia Ruskin Clinical Trials Unit, where it is relevant to my taking part in this research. I give my permission for these individuals to have access to my data.	☐
I agree to the music therapy sessions and behavioural assessments to be videotaped for monitoring and research purposes.	☐
I give my permission to be contacted again in the future to see if I am happy for video tapes taken during this study to be shared to help communicate the results of the study.	☐
I agree that data gathered in this study may be stored anonymously and securely and may be used for future research at the University of Cambridge and Anglia Ruskin University beyond the specific aims and purpose of the current study.	☐
I agree that anonymized data gathered in this study may be shared with researchers beyond the study team. This may include academic institutions, not for profit organisations or charities, or commercial companies. This does not include videotapes; videotapes will not be shared.	☐
I agree for my child and I to take part in the above study and understand that there will be no costs involved for attending the music therapy sessions.	☐

Name of parent / guardian	Date	Signature
Name of Child participant	Date	
Name of person taking consent	Date	Signature

Is the child able to give assent to participate in the study Yes ☐ No ☐

If yes, please ask child to indicate if they agree to take part, using the next page called 'giving assent'

Prof Simon Baron-Cohen
Director, Autism Research Centre
Department of Psychiatry
University of Cambridge

Dr Jonathan Pool
Senior Research Associate
Anglia Ruskin University

Giving assent

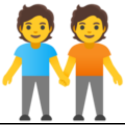	You are meeting the researchers.
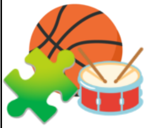	We have lots of toys. You will play with our toys with your mum or dad.
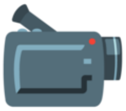	We will video it.
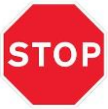	You can stop anytime.
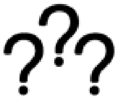	If you have any questions, please ask us.

Do you agree to take part? **Yes** ✅ ☐ **No** ❌ ☐
