# Supplementary material for: Study protocol of a randomized control trial on the effectiveness of improvisational music therapy for autistic children
Source: BMC Psychiatry. 2024 Sep 27;24:637. doi: 10.1186/s12888-024-06086-3 (PMC11437930; doi:10.1186/s12888-024-06086-3)
Supplement: Supplementary file 3 — Supplementary Material 3. [file 12888_2024_6086_MOESM3_ESM.docx]

**Supplementary material 1.** Overview of unique and essential, compatible, and proscribed principles in IMT for autistic children, and their corresponding rationale, therapist’s attitude, activity category, and examples. Originally adapted from Geretsegger, M. et al., 2015.

|  | Principle | Purpose/ Rationale | Attitude | Category of Activities | Example/ Technique |
| --- | --- | --- | --- | --- | --- |
|  | Use music | Provide a safe | Tailor and adapt | Present musical | A hello song may |
|  | making, | and reassuring | musical offerings | structures with | initially be sung |
|  | including | environment to | to the child’s likes | conviction and | on the guitar but |
|  | improvisation, | enable the child | and dislikes and | enthusiasm, | then move to the |
|  | to provide a | to feel at ease | mood of the | while adapting to | keyboard to |
|  | holding, |  | moment. Also | the needs of the | follow the child’s |
|  | reassuring and |  | make clear | child | interest. |
|  | predictable |  | endings to a |  | A structure may |
|  | structure to the |  | shared activity |  | evolve where a |
|  | sessions. Often |  | just before the |  | gathering drum is |

|  | this will involve a “Hello song” or greeting activity, and a prepared musical ending |  | child seems to be losing interest and needs to move on. |  | always played in one corner of the room, and a shared improvised piano duet is played towards the end of the session |
| --- | --- | --- | --- | --- | --- |
| Unique and  Essential | Engage the child through providing a wide range of musical styles, instruments, interactions and musical games | To motivate the child to attend, be present and interact | To use the child’s particular musical interests to widen the range of musical possibilities available to the therapist and the child | Think creatively about different ways of using music with each child | The therapist may bring in an orchestral instrument such as the clarinet to create an improvised musical exchange with the child’s recorder or reed horn playing. |
|  | To improvise and engage with the child’s movements | To enter into the child’s world and provide motivation for the child to interact | To watch, listen  and be inspired by the child | To improvise freely around the child’s movements, also incorporating interactive dance and movement exchanges | The therapist may play the keyboard to accompany the child’s walking, slowing down and stopping, to match the child’s movements. |
|  | Facilitate Musical and Emotional Attunement | Increase opportunities for awareness of self, shared attention, social reciprocity, and relationship building | Follow the child’s  focus of attention, behaviours, and interests; meet the child where they are musically and/or emotionally | Create moments of musical attunement/ synchronicity that may develop into emotional attunement/ emotional sharing; incorporate the  child’s interests and skills | Respond to the child’s utterances and behaviour using improvised music (e.g., by holding, mirroring, matching techniques) |

|  | Scaffold Interactions Dynamically | Increase opportunities for the child to comprehend, engage in, and initiate interaction | Meet the child’s initiatives/ behaviour as communicatively intended | While following the child’s lead and musical ideas, form the child’s expressions into recognisable musical forms, patterns, or motifs | Apply improvisational music therapy techniques (e.g., rhythmic grounding, shaping, exaggerating, extemporising, frameworking) |
| --- | --- | --- | --- | --- | --- |
| Unique and Essential | Using improvised music-making to create a balance between following and initiating | To provide opportunities for interaction, adaptability and negotiation. | To follow the child and enable the child to accept suggestions | The therapist moves in and out of following and initiating to reassure the child but also extend the child’s capacity to follow and initiate themselves, and develop interactive skills | The therapist accompanies the child’s rhythmic drumming on the keyboard but experiments with slight changes of tempo to encourage joint listening. If the child loses interest or focus the therapist returns to the child’s tempo. |
|  | Tap into Shared History of (Musical) Interaction | Facilitate predictability/ feeling safe and secure | Present as playful and reliable interaction partner fostering the child’s range of experience | Affirm the child’s expression and actions; jointly create musical/ social motifs and routines | Recognise and repeat the child’s expression and actions in an attuned way, giving them a musical form and value |
|  |  | Facilitate capacity for flexibility/ ability to cope with change |  | Create moments where (musical) expectations are playfully violated | Include unexpected pauses and/or dynamic variation in improvised music; redirecting techniques |
|  | Provide a Secure | Facilitate | Present as a | Provide for | Maintain similar |
|  | Environment | predictability/ | reliable and | consistency in | arrangement of |
|  |  | feeling safe and | responsible | therapy settings | objects in therapy |
|  |  | secure; keep |  |  | room and |

|  |  | the child’s anxiety low | interaction partner |  | maintain some familiarity of instruments |
| --- | --- | --- | --- | --- | --- |
|  |  |  |  | Respond to the child’s behaviour in consistent  ways | Avoid retreating from interaction partner into  “observer” role |
| Essential (but not unique to IMT) |  |  |  | Comment on/ explain interruptions and unexpected events | If it is necessary to terminate the session, explain why you find it necessary to do so |
|  | Build and Maintain a Positive Therapeutic Relationship | Enable rapport and continuation of therapy | Present with interest, respect, and confidence | Understand/ reflect upon meanings of the child’s and the therapist’s behaviour | Display empathy during (musical) interaction |
|  | Follow the Child’s Lead (Non-Directive Approach) | Facilitate intrinsic motivation | Follow the child’s focus of attention, behaviours, and interests; meet the child where they are musically and/or emotionally | Incorporate the child’s interests and motivations | Relate to a child’s preference for numbers in making up a “number song” |
|  |  | Keep the child’s anxiety low |  | Match level of session/ interaction structure to child’s needs | Allow the child to control certain aspects of the interaction; allow the child to functionalise the therapist; offer choices |
| Essential (but not unique to IMT) | Set Treatment Goals | Meet the individual child’s needs; guide and evaluate the therapy process | Enable the child to reach the respective next developmental stage in a certain skill area | Assess the child’s competences, emerging abilities, and needs; choose intervention strategies and  techniques tailored to an | If the child is aware of their own and the therapist’s (musical) actions, foster reciprocal interactions |

|  |  |  |  | assessed need in a specific area |  |
| --- | --- | --- | --- | --- | --- |
|  | Facilitate Enjoyment | Increase intrinsic motivation for interaction and opportunities for affect sharing | Present with positive affect, acceptance, affection | Incorporate the child’s interests; create pleasant and joyful atmosphere | Display interest in the child and their behaviour and joy during (musical) interaction |
|  | Adjust Setting | Facilitate | Consider the | Allow for | Improvise |
|  | According to | generalisation | whole range of | participation of | together with the |
|  | Families’ Needs, | of skills to | settings of the | parents/ | child and a family |
|  | Clinical | everyday | child’s everyday | caregivers or | member in home |
|  | Judgement, and | settings; | life | other family | environment |
|  | Practical | support families |  | members in the |  |
|  | Possibilities | in building safe |  | therapy session |  |
| Compatible |  | relationships |  |  |  |
| (but not |  | among family |  | Employ family | Discuss with |
| necessary) |  | members |  | member- | family members |
|  |  |  |  | mediated | how they can |
|  |  |  |  | intervention | employ musical |
|  |  |  |  | strategies | experiences in |
|  |  |  |  |  | interacting with |
|  |  |  |  |  | the child; |
|  |  |  |  |  | encourage |
|  |  |  |  |  | parents to sing |
|  |  |  |  |  | and vocalise |
|  |  |  |  |  | together with |
|  |  |  |  |  | their child |
